# Echocardiographic measure of dynamic arterial elastance predict pressure response during norepinephrine weaning: an observational study

**DOI:** 10.1038/s41598-021-82408-9

**Published:** 2021-02-02

**Authors:** Maxime Nguyen, Osama Abou-Arab, Stéphane Bar, Hervé Dupont, Bélaïd Bouhemad, Pierre-Grégoire Guinot

**Affiliations:** 1 Department of Anesthesiology and Intensive Care, C.H.U, Dijon, France; 2 Lipness Team, INSERM Research Center LNC-UMR1231 and LabExLipSTIC, University of Burgundy, Dijon, France; 3 Department of Anaesthesiology and Critical Care Medicine, Amiens University Hospital, 80054 Amiens, France

**Keywords:** Cardiology, Diseases, Medical research

## Abstract

The purpose of this study was to determine whether dynamic elastance E_Adyn_ derived from echocardiographic measurements of stroke volume variations can predict the success of a one-step decrease of norepinephrine dose. In this prospective single-center study, 39 patients with vasoplegic syndrome treated with norepinephrine and for whom the attending physician had decided to decrease norepinephrine dose and monitored by thermodilution were analyzed. E_Adyn_ is the ratio of pulse pressure variation to stroke volume variation and was calculated from echocardiography stroke volume variations and from transpulmonary thermodilution. Pulse pressure variation was obtained from invasive arterial monitoring. Responders were defined by a decrease in mean arterial pressure (MAP) > 10% following norepinephrine decrease. The median decrease in norepinephrine was of 0.04 [0.03–0.05] µg kg^−1^ min^−1^. Twelve patients (31%) were classified as pressure responders with a median decrease in MAP of 13% [12–15%]. E_Adyn_ was lower in pressure responders (0.40 [0.24–0.57] vs 0.95 [0.77–1.09], *p* < 0.01). E_Adyn_ was able to discriminate between pressure responders and non-responders with an area under the curve of 0.86 (CI_95%_ [0.71 to1.0], *p* < 0.05). The optimal cut-off was 0.8. E_Adyn_ calculated from the echocardiographic estimation of the stroke volume variation and the invasive arterial pulse pressure variation can be used to discriminate pressure response to norepinephrine weaning. Agreement between E_Adyn_ calculated from echocardiography and thermodilution was poor. Echocardiographic E_Adyn_ might be used at bedside to optimize hemodynamic treatment.

## Introduction

Acute circulatory failure is the result of several pathological mechanisms: hypovolemia, cardiac failure and/or vasoplegia. In the critically ill, arterial hypotension resulting from vasoplegia is a common symptom of a more complex process. Vasopressors are used to treat arterial hypotension, and they are therefore one of the main treatments for acute circulatory failure. Norepinephrine is the first-line vasopressor in intensive care^1–3^. Once the patient is stable and the underlying disease is treated, vascular function is progressively restored, and vasopressor weaning can start. Because prolonged weaning is responsible for unnecessary exposure to vasopressors (and their side effects) and because inappropriate early weaning can lead to arterial hypotension with tissue hypoperfusion, optimal vasopressor weaning is a cornerstone of hemodynamic treatment^4,5^.

Dynamic elastance is a real time indicator of the interaction between the heart and the vascular system^6^. Dynamic elastance (E_Adyn_) might be estimated as the ratio of stroke volume variation (SVV) to pulse pressure variation (PPV). Authors have demonstrated that E_Adyn_ measured by calibrated pulse contour analysis (PCA- E_Adyn_) and by uncalibrated pulse contour analysis can predict the pressure response to a decrease in norepinephrine^7–9^. Patients with acute circulatory failure and treated with vasopressors are not always monitored by calibrated pulse contour analysis. However, PPV is available for most ICU patients and SVV can be measured by echocardiography. Because the agreement between critical care echocardiography and transpulmonary dilution is moderate^10^, we aimed to determine whether E_Adyn_ calculated from echocardiographic SVV (TTE- E_Adyn_) could discriminate pressure response to a decrease in the dose of norepinephrine. Our secondary objective was to determine the agreement between E_Adyn_ measured by calibrated pulse contour analysis and E_Adyn_ measured by echocardiography.

## Results

### Baseline characteristics

Baseline characteristics are shown in Table 1. The median age was 67 [58–73] years, and 41% of patients had septic shock. Of the 39 patients included in the study, 12 were classified as pressure responders because their MAP decreased more than 10% (Supplementary file 1). The median dose of norepinephrine was 0.2 [0.09–0.38] µg.kg^−^1 min^−1^, with no difference between responders and non-responders. The median decrease in norepinephrine was 0.04 [0.03–0.05] µg.kg^−^1 min^−1^, again with no significant difference between groups (0.04 [0.03–0.04] vs 0.04 [0.04–0.05] µg.kg^−^1 min^−1^, *p* = 0.20).

**Table 1:** Baseline characteristics.

	N = 39
**Reason for norepinephrine, n (%)**	
Polytrauma	7 (18%)
Septic shock	16 (41%)
Postoperative (abdominal, cardiovascular)	16 (41%)
Body mass index (kg/m^2^)	27.4 [24.6;30.0]
Sex (men), n (%)	30 (77%)
Age (years)	67 [58;73]
Left ventricular ejection fraction (%)	50 (14)
Inotrope, n (%)	2 (5%)
Norepinephrine dose (µg/kg/min)	0.2 [0.09;0.38]

### Primary outcome

**Figure 1:**
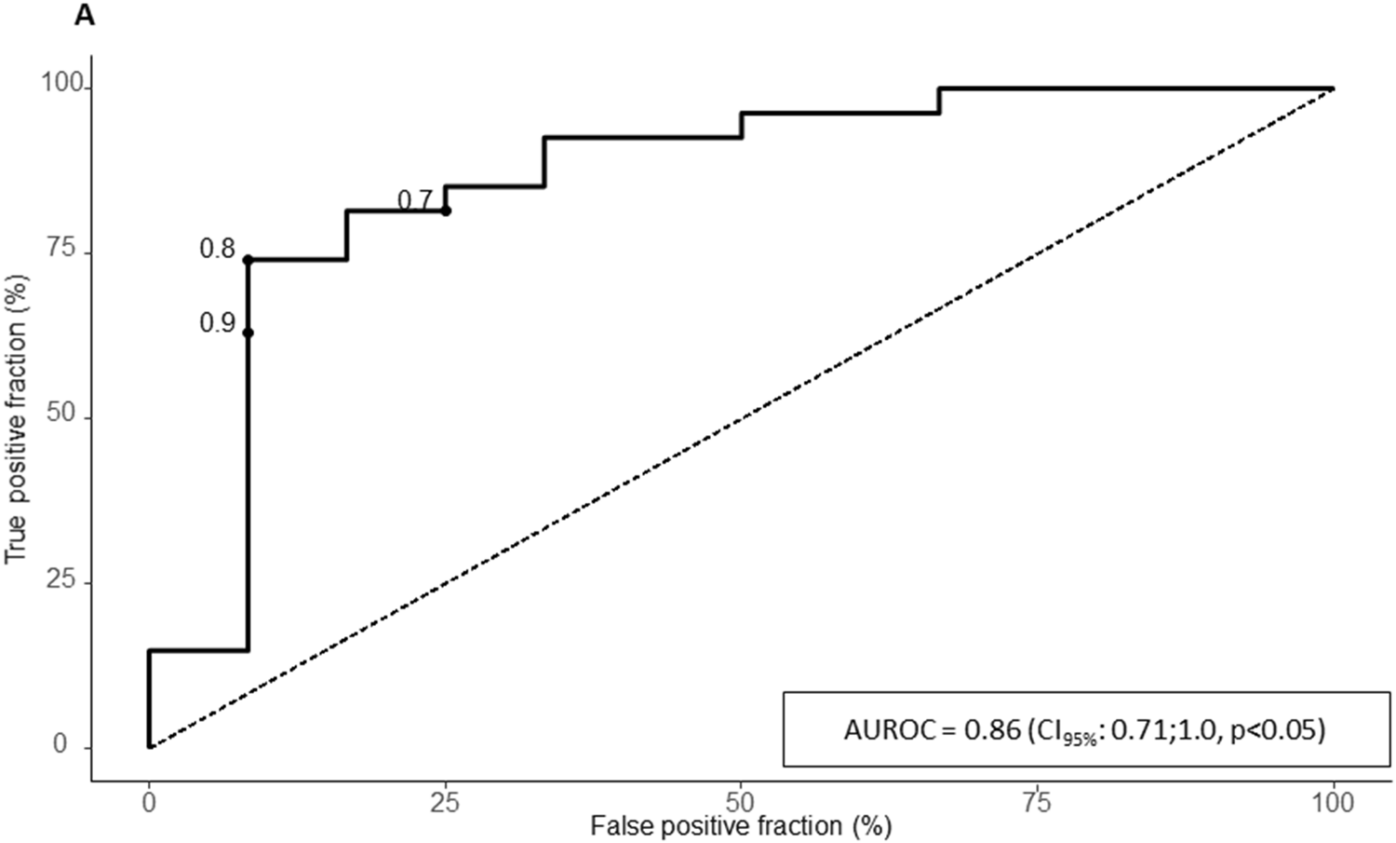
Receiving operator characteristic curve for the ability of dynamic arterial elastance to predict pressure response to a decrease of norepinephrine dose. *AUROC* area under the ROC curve.

At baseline, TTE-E_Adyn_ was lower in pressure responders (0.40 [0.24–0.57] vs 0.95 [0.77–1.09], *p* < 0.01) (Table 2), and the reduced dose of norepinephrine significantly decreased the MAP. The decrease in MAP significantly differed between groups (15% (± 5) vs 3% (± 5)). With an AUC of 0.86 (CI_95%_ [0.71 to1.0], *p* < 0.05), the TTE-E_Adyn_ predicted the decrease in arterial pressure (Fig. 1, Supplementary file 1). The optimal cut-off was of 0.80 with a sensitivity of 92% (CI_95%_ [62–100]), a specificity of 74% (CI_95%_ [54–89]), a positive likelihood ratio of 3.54, and a negative likelihood ratio of 0.11. The bounds for inconclusive TTE-E_Adyn_ were 0.47 and 0.8, and 7 patients (18%) had inconclusive TTE-E_Adyn_ values (Supplementary file 2, Table 3). The PCA-E_Adyn_ predicted the decreased in MAP with an AUC of 0.86 (CI_95%_ [0.67–0.96]). The optimal cut-off was of 0.90.

**Table 2:** Hemodynamic variable before and after decreasing norepinephrine dose.

Variable	MAP Responder N = 12	MAP Non-responder N = 27	*p*
**Norepinephrine dose (ug/kg/min)**			
Before	0.18 [0.10;0.21]	0.21 [0.09;0.42]	0.63
After	0.13 [0.06;0.17]*	0.14 [0.05;0.37]*	0.82
**Heart rate (BPM)**			
Before	85 (21)	99 (19)	0.05
After	85 (20)	98 (18)	< 0.05
**Stroke volume (mL)**			
Before	53 (19)	52 (16)	0.72
After	53 (19)	52 (16)	0.82
**Cardiac output (mL/min)**			
Before	4.6 (2.2)	5.1 (1.8)	0.48
After	4.6 (2.2)	5.1 (1.9)	0.47
**Systolic arterial pressure (mmHg)**			
Before	123 [111;128]	119 [109;127]	0.64
After	96 [94;106]*	114 [107;122]*	< 0.01
**Diastolic arterial pressure (mmHg)**			
Before	57 (9)	59 (9)	0.46
After	50 (5)*	57 (8)*	< 0.01
**Mean arterial pressure (mmHg)**			
Before	78 (8)	79 (9)	0.61
After	66 (6)*	77 (8)*	< 0.01
**Central venous pressure (mmHg)**			
Before	12 [11;13]	11 [7;14]	0.93
After	11 [10;12]	11 [7;13]*	0.88
**Dynamic elastance (echography)**			
Before	0.40 [0.24;0.57]	0.95 [0.77;1.09]	< 0.01
After	0.31 [0.26;0.71]	0.76 [0.55;1.09]	0.03
**Stroke volume variation (echography)**			
Before	25 [15;37]	13 [9;19]	0.02
After	22 [14;29]	15 [9;24]	0.13
**Pulse pressure variation**			
Before	8 [5;16]	12 [8;16]	0.30
After	9 [6;15]	13 [8;17]	0.39

*p* value refer to between group comparison, * represent significant differences for within group (before-after) comparison.

MAP: mean arterial pressure.

**Table 3:** Tests performance of echographic E_Adyn_ to predict mean arterial pressure decrease higher than 10% after norepinephrine decrease for various threshold.

	E_Adyn_ < 0.47	E_Adyn_ < 0.8
Sensitivity	0.67 [0.35; 0.90]	0.92 [0.62; 1.00]
Specificity	0.93 [0.76; 0.99]	0.74 [0.54; 0.89]
Positive predictive value	0.80 [0.44; 0.97]	0.61 [0.36; 0.83]
Negative predictive value	0.86 [0.68; 0.96]	0.95 [0.76; 1.00]
Positive likelihood ratio	9.00 [2.24;36.2]	3.54 [1.83; 6.84]
Negative likelihood ratio	0.36 [0.16; 0.81]	0.11 [0.02; 0.74]

### ***Agreement between TTE-E***_***Adyn***_*** and PCA-E***_***Adyn***_

**Figure 2:**
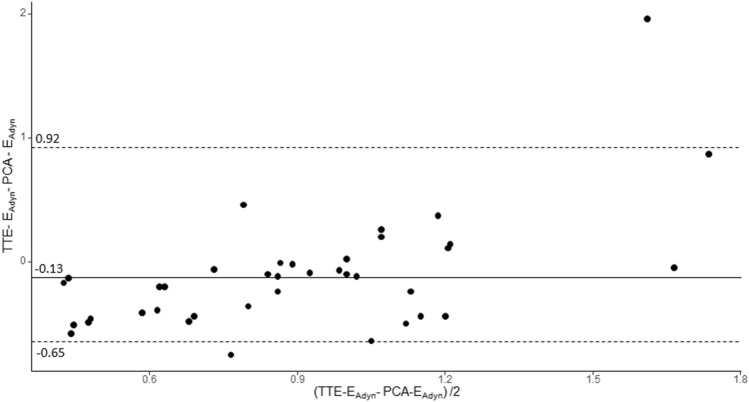
Bland–Altman plots for the measure of dynamic arterial elastance from echocardiography and from pulse contour analysis (transpulmonary thermodilution). The plain lane represents the median, dashed lines represent the 2.5th and 97.5th percentiles. *TEE-*_*EAdyn*_ dynamic elastance calculated from transthoracic echocardiography, *PCA-*_*EAdyn*_ dynamic elastance calculated from pulse contour analysis.

The agreement between TTE-E_Adyn_ and PCA-E_Adyn_ is presented in Fig. 2. The median bias was − 0.13 [− 0.39 to − 0.06] with the limit of agreement between − 0.65 and 0.92 (Fig. 2). The concordance correlation coefficient was poor (0.31 [0.13–0.48]).

## Discussion

The results of this study suggest that E_Adyn_ calculated from SVV measured by echocardiography can be used to discriminate pressure response to a decrease in norepinephrine dose. However, we found that there was poor agreement between TTE-E_Adyn_ and PCA-E_Adyn_ despite good ability to predict pressure response.

E_Adyn_ is usually obtained with an invasive hemodynamic monitoring system, but such devices may be unavailable in our patients. On the contrary, point-of-care ultrasound is an extensively used and non-invasive tool^11^. Thus, extending the validity of E_Adyn_ to echocardiographic measurements means that more patients could benefit from this indicator. Ultrasound is an operator-dependent technique with lower reproducibility than hemodynamic devices. In addition, studies have highlighted inconsistencies between thermodilution and ultrasound for cardiac output monitoring^12^. In the present cohort, the agreement between echocardiographic E_Adyn_ and E_Adyn_ calculated by thermodilution was low. The bias was negative, meaning that SVV was overestimated by echocardiography. This observation explains why our cut-off was lower than those described by thermodilution^7^. Our observations are in line with those of De Castro et al., who demonstrated poor agreement between SVV measured with pulse contour analysis and aortic Doppler^13^. This low agreement may be explained by several factors: the calculation of SV_max_ and SV_min_ with echocardiography was manually performed, whereas those calculated by EV100 system may automatically measure, and the time sample to calculate the SVV with the EV1000 system may differ of this of echocardiography.

In practice, E_Adyn_ is an easy-to-read indicator that could be used by the physician to understand the arterial load and its coherence with cardiac status (i.e. ventriculo-arterial coupling) and thus to determine which treatments should be used or withdrawn. High E_Adyn_ might indicate an inappropriately high arterial load compared with stroke volume. According to the concept of ventriculo-arterial coupling, stroke work is optimized when the ventricular and arterial systems are coupled^14^. Because of its action on both α and β adrenergic receptors^15^, norepinephrine has an effect on arterial load, cardiac preload and /or cardiac contractility^16^. Norepinephrine might therefore be associated with both preload and non-preload stroke volume changes^17^. In patients with high E_Adyn_, lowering arterial load by decreasing norepinephrine might improve ventriculo-arterial coupling. The optimization of stroke work could compensate for the drop in arterial vasoconstriction, explaining the good tolerance (MAP non-response) to norepinephrine weaning. The method developed by Chen to obtain E_Adyn_ requires complex calculation^18^ and simplified methods are not reliable in the ICU^19^; these limits suggest that bedside measurement of E_Adyn_ may be more clinically appropriate than the measurement of ventriculo-arterial coupling. Recently, Monge Garcia et al*.* have demonstrated that Ea_dyn_ was inversely related to ventriculo-arterial coupling^6^, and that Ea_dyn_ could be used to track changes of ventriculo-arterial coupling following hemodynamic treatment.

We acknowledge that this study has some limitations. First, the small sample size limits the power of the analysis, however, the results are significant and the sample size is in accordance with previous studies^7,20,21^. The sample size also limited the use of parametric approach and prevented the realisation of more specific analysis such as ROC curve comparison between TTE-E_Adyn_ and PCA-E_Adyn_ (or TTE-SVV and PCA-SVV). The monocentric nature of the study limits the external validity, but our results are in accordance with previously reported data^7–9^. Finally, we did not measure ventriculo-arterial coupling.

## Conclusion

E_Adyn_ calculated from the echocardiographic estimation of the SVV and the invasive arterial PPV could discriminate pressure response to norepinephrine decrease. Despite the poor agreement between SVV measurements obtained by thermodilution and by echocardiography, E_Adyn_ might be useful at the patient’s bedside to optimize vasopressive treatment.

## Methods

### Patients

This is an ancillary study using data from a prospective observational cohort. The study objectives and procedures were approved by the local ethics committee (*Comité de Protection des Personnes Nord-Ouest II* CHU—Place V. Pauchet, 80054 AMIENS Cedex 1). All subjects or next of kin (closest parent, legal guardian) if the patient was unable to be informed, received written information about the study and provided their informed consent to participate. The study was performed in accordance with the ethical standards of the 1964 Declaration of Helsinki. This prospective, observational study was conducted in the intensive care unit (ICU) of the Amiens University Hospital between July 2016 and March 2017. We included patients with a diagnosis of vasoplegic syndrome, treated with norepinephrine, and for whom the attending physician decided to decrease the norepinephrine dosage. Patients treated with epinephrine and/or dobutamine, and patients with arrhythmia, intra-abdominal hypertension or younger than 18 years old were excluded.

### Hemodynamic parameters

All patients were monitored with arterial catheterisation, central venous pressure (CVP) *and transpulmonary thermodilution* (EV1000 system, EDWARDS, Irvine CA, USA). Transthoracic echocardiography (CX50 Ultrasound System and an S5-1 Sector Array Transducer, PHILIPS MEDICAL SYSTEM, Suresnes, France) was performed by a physician with advanced echocardiography certificate. Left ventricular ejection fraction (LVEF) was calculated using Simpson’s method on a four-chamber view. The diameter of the left ventricular outflow tract (LVOT) was measured on a long-axis parasternal view at the time of patient inclusion. The LVOT velocity–time integral (VTI_LVOT_) was measured with pulsed Doppler on a five-chamber apical view. Stroke volume (SV; mL) was calculated as VTIAo × aortic area. Cardiac output (CO) (l/min) was calculated as SV × heart rate (HR). Respiratory variation of SV (SVV_TTE_) was calculated as: SVV_TTE_ = (SV_max_–SV_min_)/((SV_max_ + SV_min_)/2))*100. The maximum and minimum SV values were identified during one respiratory cycle (expiratory/inspiratory times), and averaged over 5 cycles to obtain SV_max_ and SV_min_^22,23^. We calculated the reproducibility of SVV_TTE_ that was 8.1 (± 4.4) %. The physician who performed echocardiography was blinded to the results of the study.

PPV was automatically calculated by the Philips monitoring system, representing the average of five successive values. SVV and PPV were sampled during the same time period on a radial arterial line^13^. We measured SVV with a calibrated pulse contour analysis (EV1000, Edwards Life science Irvine, software version 4.0), representing the average of three successive values. Echocardiographic dynamic arterial elastance (TTE-E_Adyn_) was calculated as the ratio of PPV/SVV_TTE_, and calibrated pulse contour analysis dynamic arterial elastance (PCA-E_Adyn_) was calculated as PPV/SVV.

### Study procedures

The intervention assessed in this study was a one-step decrease in the dose of norepinephrine. The following clinical parameters were recorded for each patient: age, gender, surgical/medical history, and indications for norepinephrine treatment. Hemodynamic variables including HR, systolic arterial pressure (SAP), mean arterial pressure (MAP), diastolic arterial pressure (DAP), central venous pressure (CVP), SVV_TTE_, SVV_,_ PPV, and cardiac output (CO) were recorded at baseline. The dose of norepinephrine was then decreased. After the hemodynamic variables had stabilised, defined as < 10% variation in MAP over a 30-min period, a second set of measurements (HR, SAP, MAP, DAP, CVP, PPV, SVV, SVV_TTE_ CO) was recorded. Ventilator settings and sedation were kept constant throughout the study period. All patients had mechanical ventilation in volume-controlled mode. Ventilator settings (oxygen inspired fraction, tidal volume, respiratory rate and end positive pressure) were not modified during the study period.

### Statistical analyses

Retrospective power analysis shows that a sample of 39 patients enable to demonstrate an area under the receiver-operating-characteristic curve greater than 0.77, a power of 80%, and an α risk of 0.05. The distribution of variables was assessed using a Shapiro–Wilk test. Data are expressed as numbers, proportions (in percent), medians [interquartile range] or as means (standard deviation). The coefficient of variation (CV), precision and least significant change (LSC) for MAP were calculated on the overall population. The LSC was 7% (confidence interval 95% (CI_95%_): 4–10). Based on LSC, we defined a positive response (pressure responders) as a decrease in MAP of over 10%^8^. Qualitative data were compared with a chi-squared test or Fisher’s test, and quantitative data were assessed with a Student’s *t *test or Kruskal–Wallis sum rank test, as appropriate. Paired data were compared with paired Student’s *t *test or Wilcoxon signed rank test. Changes in hemodynamic variables were expressed as percentages from baseline value. Receiving operator characteristic (ROC) curves were drawn and the areas under the curve (AUC) were calculated. The optimal cut-point was determined using the Youden Method. The concordance correlation coefficient between TTE-E_Adyn_ and PCA-E_Adyn_ was calculated, and the agreement was represented on a Bland–Altman plot. A grey-zone approach was used, and values for which both sensitivity and specificity were lower than 90% were considered inconclusive. The threshold for statistical significance was set at *p* < 0.05. RSTUDIO (Version 1.1.447-2009-2018 RSTUDIO, Inc.) was used for all statistical analyses.

### Ethics approval and consent to participate

This is an ancillary study using data from a prospective observational cohort. The study objectives and procedures were approved by the local ethics committee (*Comité de Protection des Personnes Nord-Ouest II* CHU—Place V. Pauchet, 80054 AMIENS Cedex 1). All subjects or next of kin (closest parent, legal guardian) if the patient was unable to be informed, received written information about the study and provided their informed consent to participate.

## Supplementary Information

Supplementary Information

## Data Availability

All relevant data are within the paper. Raw data are available after notification and authorization of the competent authorities. In France, all computer data (including databases, particular patient data) are protected by the National Commission on Informatics and Liberty (CNIL), the national data protection authority for France. CNIL is an independent French administrative regulatory body whose mission is to ensure that data privacy law is applied to the collection, storage, and use of personal data. As the database of this study was authorized by the CNIL, we cannot make available data without prior agreement of the CNIL. Requests may be sent to: elisabeth.laillet@chu-dijon.fr.

## Abbreviations

AUC: Area under the curve
CI: Confidence interval
CO: Cardiac output
CV: Coefficient of variation
CVP: Central venous pressure
DAP: Diastolic arterial pressure
E_Adyn_: Dynamic arterial elastance
HR: Heart rate
LSC: Least significant change
LVEF: Left ventricular ejection fraction
LVOT: Left ventricular outflow tract
MAP: Mean arterial pressure
PCA: Pulse contour analysis
PPV: Pulse pressure variation
ROC: Receiving operator characteristic
SAP: Systolic arterial pressure
SV: Stroke volume
SVV: Stroke volume variation
TTE: Transthoracic Echocardiography
VTI_LVOT_: Left ventricular outflow tract velocity–time integral

## References

[1] Rhodes A (2017). Surviving sepsis campaign: international guidelines for management of sepsis and septic shock: 2016. Intensive Care Med..

[2] Thiele H, Ohman EM, Desch S, Eitel I, de Waha S (2015). Management of cardiogenic shock. Eur. Heart J..

[3] Duranteau J (2015). Recommandations sur la réanimation du choc hémorragique. Anesth. Réanimation.

[4] Boissier F (2017). Left ventricular systolic dysfunction during septic shock: the role of loading conditions. Intensive Care Med..

[5] Lamontagne F (2018). Pooled analysis of higher versus lower blood pressure targets for vasopressor therapy septic and vasodilatory shock. Intensive Care Med..

[6] Monge García MI (2020). Dynamic arterial elastance as a ventriculo-arterial coupling index: an experimental animal study. Front. Physiol..

[7] Guinot P-G, Bernard E, Levrard M, Dupont H, Lorne E (2015). Dynamic arterial elastance predicts mean arterial pressure decrease associated with decreasing norepinephrine dosage in septic shock. Crit. Care.

[8] Bar S (2018). Dynamic arterial elastance measured by uncalibrated pulse contour analysis predicts arterial-pressure response to a decrease in norepinephrine. Br. J. Anaesth..

[9] Guinot P-G (2017). Monitoring dynamic arterial elastance as a means of decreasing the duration of norepinephrine treatment in vasoplegic syndrome following cardiac surgery: a prospective, randomized trial. Intensive Care Med..

[10] Vignon P (2018). Hemodynamic assessment of patients with septic shock using transpulmonary thermodilution and critical care echocardiography: a comparative study. Chest.

[11] Zieleskiewicz L (2015). Point-of-care ultrasound in intensive care units: assessment of 1073 procedures in a multicentric, prospective, observational study. Intensive Care Med..

[12] Zhang Y (2019). Ultrasound cardiac output monitor and thermodilution for cardiac function monitoring in critical patients: a Meta-analysis. Zhonghua Wei Zhong Bing Ji Jiu Yi Xue.

[13] De Castro V (2006). Comparison of stroke volume (SV) and stroke volume respiratory variation (SVV) measured by the axillary artery pulse-contour method and by aortic Doppler echocardiography in patients undergoing aortic surgery. Br. J. Anaesth..

[14] Sunagawa K, Maughan WL, Sagawa K (1985). Optimal arterial resistance for the maximal stroke work studied in isolated canine left ventricle. Circ. Res..

[15] Zimmerman, J., Lee, J. P. & Cahalan, M. Vasopressors and Inotropes. in *Pharmacology and Physiology for Anesthesia* 520–534 (Elsevier, 2019). 10.1016/B978-0-323-48110-6.00025-9.

[16] Annane D (2018). A global perspective on vasoactive agents in shock. Intensive Care Med..

[17] Guinot P-G, Longrois D, Kamel S, Lorne E, Dupont H (2018). Ventriculo-arterial coupling analysis predicts the hemodynamic response to norepinephrine in hypotensive postoperative patients: a prospective observational study. Crit. Care Med..

[18] Chen C-H (2001). Noninvasive single-beat determination of left ventricular end-systolic elastance in humans. J. Am. Coll. Cardiol..

[19] Nguyen M (2019). Agreement between different non-invasive methods of ventricular elastance assessment for the monitoring of ventricular–arterial coupling in intensive care. J. Clin. Monit. Comput..

[20] Monge Garcia MI, Gil Cano A, Gracia Romero M (2011). Dynamic arterial elastance to predict arterial pressure response to volume loading in preload-dependent patients. Crit. Care.

[21] Huette P, Abou-Arab O, Longrois D, Guinot PG (2020). Fluid expansion improve ventriculo-arterial coupling in preload-dependent patients: a prospective observational study. BMC Anesthesiol..

[22] Guinot PG (2014). Respiratory stroke volume variation assessed by oesophageal Doppler monitoring predicts fluid responsiveness during laparoscopy. Br. J. Anaesth..

[23] Guinot PG (2013). Ability of stroke volume variation measured by oesophageal Doppler monitoring to predict fluid responsiveness during surgery. Br. J. Anaesth..

