# Supplementary material for: Echocardiographic measure of dynamic arterial elastance predict pressure response during norepinephrine weaning: an observational study
Source: Sci Rep. 2021 Feb 2;11:2853. doi: 10.1038/s41598-021-82408-9 (PMC7854654; doi:10.1038/s41598-021-82408-9)
Supplement: Supplementary file 1 — Supplementary Information [file 41598_2021_82408_MOESM1_ESM.docx]

**Echocardiographic measure of dynamic arterial elastance predict pressure response during norepinephrine weaning: an observational study.**

Maxime Nguyen^1-2,*^, Osama Abou-Arab^3^, MD, PhD, Stéphane Bar^3^, MD, PhD, Hervé Dupont^3^, MD, PhD, Bélaïd Bouhemad^1-2^, MD, PhD, Pierre-Grégoire Guinot^1-2^, MD, PhD

**Supplementary data**

**
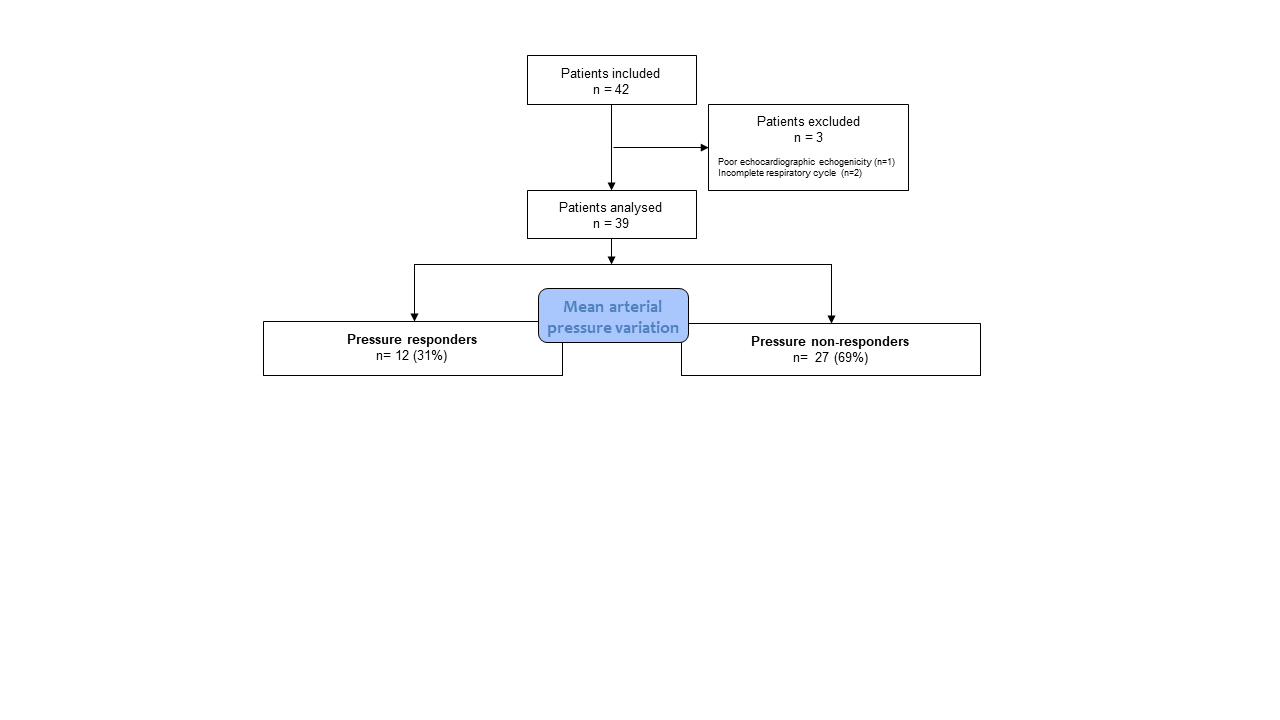
**

**Supplementary file 1.** Flow chart of the study

Pressure response is defined as a decrease in MAP of over 10%.

**
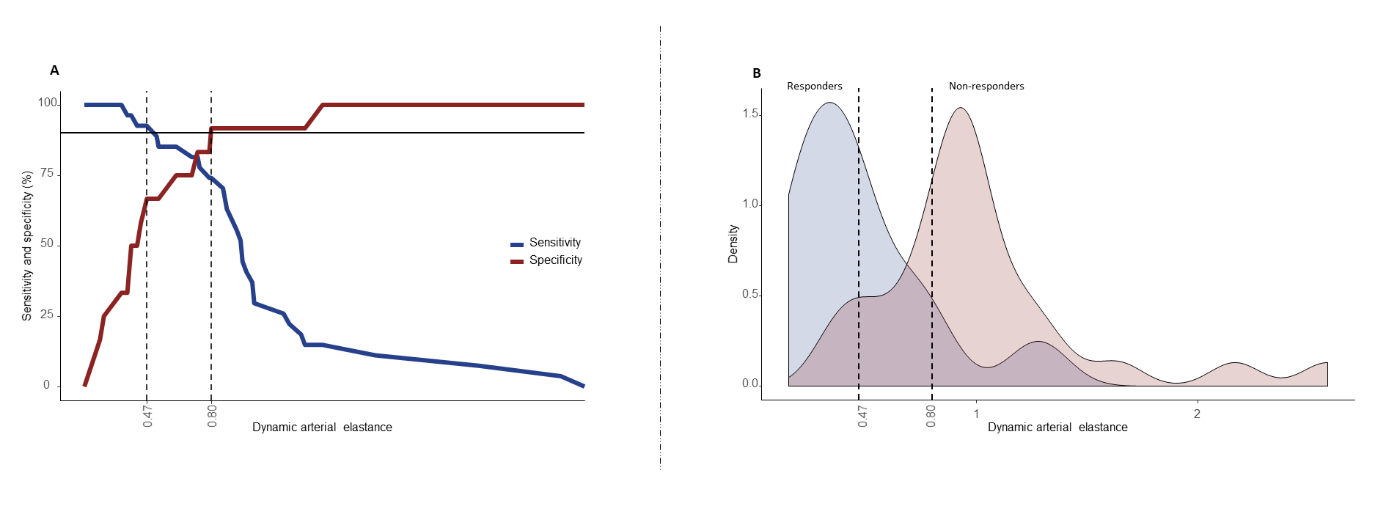
**

**Supplementary file 2.** Grey zone approach

**A**: Sensitivity and specificity of dynamic arterial elastance for the ability of dynamic arterial elastance to predict pressure response to a decrease of norepinephrine dose.

The plain line represents 90%

Dashed lines represent thresholds for dynamic arterial elastance value with sensitivity or specificity over 90%

**B**: Density plot for dynamic arterial elastance depending on pressure response.

Dashed lines represent thresholds for dynamic arterial elastance value with sensitivity or specificity over 90%
